# Evaluation of Metabolic and Cardiovascular Risks in patients with Prediabetes: Cross-sectional study

**DOI:** 10.12669/pjms.41.11.12690

**Published:** 2025-11

**Authors:** Ahmed M Ahmed, Mohammed A Alsarani, Ahmed M Alkhattabi, Ahmad A Aljohani, Fahad S Aljohani, Abdulaziz J Aldubiani

**Affiliations:** 1 Ahmed M Ahmed Department of Clinical Laboratory Sciences, College of Applied Medical Sciences, Taibah University, Al-Madinah, Kingdom of Saudi Arabia; 2 Mohammed A Alsarani Medical Laboratory Specialist, Medicine College, Taibah University, Al-Madinah, Kingdom of Saudi Arabia; 3 Ahmed M Alkhattabi Medical Laboratory Specialist, Medicine College, Taibah University, Al-Madinah, Kingdom of Saudi Arabia; 4 Ahmad A Aljohani Medical Laboratory Department, College of Applied Medical Sciences, Taibah University, Yanbu, Saudi Arabia; 5 Fahad S Aljohani Medical Laboratory Specialist, Medicine College, Taibah University, Al-Madinah, Kingdom of Saudi Arabia; 6 Abdulaziz J Aldubiani RN Nurse technician, Medicine College, Taibah University, Al-Madinah, Kingdom of Saudi Arabia

**Keywords:** Cardiovascular Risk, HbA1c, Prediabetes, Pre-D, T2DM

## Abstract

**Objective:**

This study aimed to evaluate the metabolic and cardiovascular risk of patients with prediabetes (pre-D) compared to type two diabetes mellitus (T2DM) patients and healthy subjects, and to predict cardiovascular risk associated with glycemic disturbance.

**Methodology:**

In this cross-sectional study, eighty patients with prediabetes were compared with ninety T2DM and one hundred healthy individuals as controls enrolled from the University Medical Center, Taibah University, Madinah, Kingdom of Saudi Arabia, from 23 June 2024 to 06 February 2025. Biochemical (FPG, HbA1c, insulin, and lipid profiles), anthropometric (BMI, WC, F%, FM, and FFM), and clinical (blood pressure, smoking, and education status) profiles were measured.

**Results:**

Pre-D and T2DM groups had significantly higher BMI, F%, FM, FPG, HbA1c, insulin, and TG than controls (p <0.01). Regression analysis showed that BMI, F%, FM, FPG and insulin are independent predictors of pre-D risk (p <0.001); moreover, crosstabs revealed the assessment of cardiovascular risk which revealed significant high odds ratios in pre-D subjects with hypertension (OR = 31.7), hyperglycemia (OR = 40), metabolic syndrome (OR = 35), smoking (OR = 55) and obesity (OR = 64.7).

**Conclusion:**

Pre-D exhibited significant metabolic alterations and higher risk for cardiovascular disease, which necessitates early screening and treatment. Factors like smoking and high blood pressure increase cardiovascular risk, while factors related to adiposity and insulin resistance influence glycemic levels.

## INTRODUCTION

Prediabetes (pre-D) is considered a metabolic disorder characterized by increased levels of blood sugar that are insufficient to confirm diagnosis of T2DM. It contains the impaired fasting glucose, increased glycated hemoglobin (HbA1c), and impaired glucose tolerance. Globally, the incidence rate of pre-D is rising; it is estimated that more than 720 million people suffered from it in 2021 and the rate expected to elevate by 2045 to one billion.^1^

Pre-D is public health issue concern in Saudi Arabia, with adult prevalence rates between 20% and higher than 25%. Urbanization-related lifestyle such as obesity, unhealthy eating, and inactivity are associated with this worrying trend. Based on the heart health promotion study, those with prediabetes have a 2.64-fold elevated risk of cardiovascular disease compared to those with normal blood sugar levels. These findings emphasize the urgent need for comprehensive screening and prevention.^2^

New findings suggest that prediabetes is not just a temporary stage but is linked to considerable health hazards. A large study in the United States indicates that people with pre-D were more likely to have acute coronary syndrome, ischemic stroke and heart failure compared to people without pre-D.^3^ In same manner, a meta-analysis revealed that pre-D associated with risk of overall mortality and CVD.^4^

The association between pre-D and metabolic syndrome (MetS) aggravate health risks. According to study in Thailand, about 54.3% of individuals who look healthy had pre-D and a significant number of this group met the MetS criteria, including elevated waist circumference, high blood pressure and dyslipidemia.^5^ In Turkey, study showed that 78.4% of pre-D individuals also experienced MetS, with elevated rates of hypertension, abdominal obesity and dyslipidemia relative to those without MetS.^6^

Prediabetes is associated with serious health risks particularly with CVD and MetS; early detection and intervention are so critical. This study was undertaken to evaluate the anthropometric, biochemical and cardiovascular risk of individuals with pre-D, compared to healthy and T2DM subjects, as well as to identify early signs of glycemic progression.

## METHODOLOGY

In this cross-sectional research, eighty individuals with pre-D and ninety individuals with T2DM compared with hundred healthy participants (controls). Populations were included from the University Medical Center, Taibah University, Madinah, Saudi Arabia from 23/6/2024 – 06/2/2025. Prediabetes and T2DM patients were diagnosed based on WHO criteria: HbA1c levels of 5.7-6.4% for prediabetes and HbA1C ≥ 6.5% and fasting blood glucose (FBG) ≥7.0 mmol/L for T2DM.^7^ Patients with acute illnesses, cancer, pregnancy, or liver disorders were excluded, patients with type 1 diabetes mellitus excluded after differentiating with a set of clinical signs (adult onset, insulin resistance, and overweight/obesity), autoimmunity absence, and response to oral medications. The sociodemographic information including age, sex and educational attainment and the details on tobacco consumption and information on hypertension were gathered through the World Health Organization (WHO) stepwise strategy for chronic disease risk factor surveillance instrument.

### Ethical approval:

The “Research Ethics Committee” of Taibah University approved the study protocol (Approval No. 2024/119/161/MLT; dated: June 19, 2024). The committee functions in line with the “1964 Declaration of Helsinki and its revisions”. Informed consent in writing was secured from participants before they took part in the study.

### Anthropometric profiles:

The measurements of body mass index (BMI), height (Ht), weight (Wt), waist circumference (WC), body water (BW), fat percentage (F%), fat mass (FM) and free fat mass (FFM) were conducted with the InBody TANITA BC-418, a non-invasive device using Bioelectrical Impedance Analysis (BIA). (TANITA Company, Japan).

### Biochemical profile:

Fasting plasma glucose (FPG), HbA1c, insulin, total cholesterol (TC), triglycerides (TG), HDL and LDL were measured with the Cobas c311 Autoanalyzer.

### Statistical analysis:

The analysis was conducted using SPSS version 23 (IBM Corporation, Armonk, NY, United States). ANOVA (Tukey’s post hoc analysis), unpaired t-test and Pearson correlation were utilized when appropriate. Chi-squared/Fisher’s used for frequencies. Regression models and crosstabs were employed to examine the independent impact of prediabetes on developing CVD risks. A P<0.05 indicates significant differences.

The **demographic,** anthropometric, and biochemical profiles of participants are shown in Table-I. There is no notable difference in age, gender and smoking status among participants. Education status among study groups reveals a significant difference (p<0.05) with higher number of pre-D individuals. In addition, BMI, Wt, F%, FM and FFM exhibited significant differences between the groups, with higher values in the pre-D and T2DM (p<0.01). Moreover, FPG and HbA1c levels were elevated in the pre-D and T2DM groups compared to controls (p<0.01) and additionally, FPG and HbA1c were higher in T2DM than in the pre-D group (p<0.01).

**Table-I:** Demographic, anthropometric and biochemical data of participants.

	Control n=100	Pre-D n=80	T2DM n=90	P value
Age (years)	42±11.3	43.6±8.8	45.1±9.2	0.11
*Gender*				
- Male	63	45	56	0.83
- Females	37	35	34	
*Education level:*				
- None-school	4	9	7	0.03
- School	53	36	56	
- University	43	35	27	
*Smoking*				
- Smoker	28	24	19	0.53
- None-smoker	72	56	71	
BMI	27.8±4.8	31.1±4.3	30.9±7.6	<0.001
Ht	165.3±9.5	166.9±7.9	164.7±8.9	0.32
Wt	79.7±19.6	87.7±13.8	85.9±16.5	0.007
WC	92.3±25.8	96.2±18.9	95.5±31.3	0.59
F%	31.2±8.1	37.9±9.1	36.6±8.1	<0.001
FM	25.6±10.3	35.6±13.1	33.6±10.5	<0.001
FFM	50.7±10.3	56.1±10.6	55.2±8.7	0.001
BW	41.8±7.7	40.3±7.7	39.3±9	0.11
SBP	130.4±21.4	136.1±20.8	133.1±42.2	0.51
DBP	78.3±13.8	82.8±10.3	81.2±19.3	0.17
FPG (mmol/l)	5.3±0.4	6.2±0.4	8.9±3.3	0.001
HbA1c (%)	5.2±0.36	5.9±0.18	7.9±1.9	0.001
Insulin (µU/ml)	14.1±8.5	20.3±10.1	16.9±13.1	0.002
TC (mmol/l)	5.08±0.89	5.23±1.33	5.38±1.07	0.16
TG (mmol/l)	1.29±0.73	1.51±0.78	1.74±0.88	0.001
HDL (mmol/l)	1.29±0.32	1.19±0.25	1.28±0.36	0.16
LDL (mmol/l)	3.15±0.80	3.33±1.01	3.31±1.01	0.38

***Pre-D:** prediabetes. **T2DM:** type two diabetes mellitus. **P:** probability. **BMI:** body mass index. **Ht:** height. **Wt:** weight. **WC:** waist circumference. **F%:** fat percentage. **FM:** fat mass. **FMM:** free fat mass. **BW:** body water. **SBP:** systolic blood pressure. **DBP:** diastolic blood pressure. **FPG:** fasting plasma glucose. **HbA1c:** hemoglobin A1c. **TC:** total cholesterol. **TG:** triglyceride. **HDL:** high-density lipoprotein. **LDL:** low-density lipoprotein.*

Comparison of biochemical profiles with different characteristics in pre-D group is shown in Table-II. Participants with no school exhibited a notably increase in HbA1c, TC and LDL levels (p<0.05). Moreover, smokers exhibited increased LDL levels (p<0.05). Furthermore, hypertensive individuals exhibited considerably elevation in SBP and DBP (p<0.01).

**Table-II:** Comparison of biochemical parameters in different characteristics in pre-D subjects.

	Gender		Education level			Smoking		Blood pressure	
	Female n=35	Male n=45	None-school n=9	School n=36	University n=35	None-smoker n=56	Smoker n=24	Hypertensive n=38	None-hypertensive n=42
FPG	114.8±12.7	110.5±7.2	115±7.8	111.4±79.7	112.2±67.1	112.1±7.7	113.1±65.1	111.9±8.3	113±68.5
HbA1c	6±0.3	5.9±0.2	6.1±0.27	5.9±0.15	5.9±0.16	5.9±0.16	6±0.25	5.9±0.18	5.9±0.19
Insulin	21±8.8	19.8±10.9	22.5±13.1	22.6±11.3	17.1±6.3	20.6±10.7	19.3±7.6	23±11.6	17.2±11.6
TC	219.1±68.7	191±41.5	263.4±89.9	191.5±34.2	192.5±32.7	197.2±52.3	220.7±47.8	208.6±69.8	196.1±31.2
TG	150.8±77.1	121.8±62.1	197.8±113.1	114.9±75.2	130.6±90.3	133.5±68.3	135.1±76.2	148.7±81.7	124.7±56.8
HDL	50.1±11.1	43.6±18.2	49.8±13.6	49±10.1	42.3±16.9	45.1±8.7	50.3±12.9	47.8±9.2	44.3±9.9
LDL	135.6±49.9	124.1±29.3	171.3±63.1	119.5±29.1	123.4±27.8	125.8±21.9	138.9±17.8	130.6±52.5	125.8±24.5

The correlation between glycemic and insulin with anthropometric and biochemical profiles in pre-D, FPG correlates positively with F% and HbA1c (p<0.05) and negatively with FFM (p<0.01). Moreover, insulin strongly correlates with BMI, F% and FM (p<0.01) and with HDL (p<0.05) is represented in Table-III. Table-IV shows the regression analysis of variables linked to the risk of developing diabetes in pre-D group in relation to HbA1c. It identifies significant positive predictors for BMI (β=0.508, p<0.001), F% (β=0.415, p=0.001), FM (β=0.503, p<0.001), FPG (β=0.269, p<0.001) and Insulin (β=0.443, p<0.001).

**Table-III:** Correlation between glycemic and insulin with anthropometric and biochemical profiles.

	Age	BMI	WC	F%	FM	FFM	FPG	HbA1c	Insulin	TC	TG	HDL	LDL
FPG	0.24	0.12	-0.05	0.2	0.1	-0.3	-	0.2	0.1	-0.04	0.1	-0.09	-0.03
HbA1c	-0.05	0.01	-0.04	0.1	0.1	-0.1	0.2	-	0.1	0.01	0.06	0.1	-0.03
Insulin	-0.2	0.59	-0.03	0.48	0.6	0.2	0.1	0.1	-	0.02	0.04	0.2	-0.1

**Table-IV:** Regression analysis of variables associated with the risk for diabetes development against HbA1c.

	Standardized Coefficient	SE	Odd ratio	95% CI		P value
	Beta			lower	Upper	
Age	-0.181	0.119	6.13	5.89	6.37	0.16
BMI	0.508	0.116	5.45	5.22	5.568	<0.001
WC	0.131	0.122	5.84	5.60	6.09	0.32
F%	0.415	0.093	5.65	5.46	5.84	0.001
FM	0.503	0.06	5.72	5.60	5.83	<0.001
FFM	0.116	0.128	5.85	5.60	6.11	0.37
FPG	0.269	0.351	5.22	4.52	5.92	<0.001
Insulin	0.443	0.048	5.80	5.70	5.90	<0.001
BW	0.071	0.130	5.89	5.63	6.15	0.58
SBP	0.105	0.185	5.83	5.46	6.21	0.46
DBP	0.182	0.221	5.69	5.24	6.13	0.20
TC	-0.114	0.096	6.05	5.85	6.24	0.38
TG	-0.053	0.052	5.98	5.88	6.09	0.68
HDL	0.160	0.112	5.83	5.60	6.05	0.22
LDL	-0.188	0.081	6.08	5.92	6.24	0.15

Estimate of cardiovascular development risks in the pre-D group via cross tabulation are shown in Table-V. Hyperglycemia: OR = 40 (CI: 7.12-224.9), Obesity: OR = 64.7 (CI: 7.4-565.9), Metabolic Syndrome: OR = 35 (CI: 6.1-199.9), Smoking: OR = 55 (CI: 8.8-343.2) and Hypertension: OR = 31.7 (CI: 6.16-162.6). These factors collectively were strongly independently linked to the risk of developing CVD (p<0.01).

**Table-V:** Odds Ratio for factors affect CVD development risk estimates in pre-D group versus non-cardiovascular cohorts using cross tabulation.

	Value	95% CI		P value
		Lower	Upper	
Hyperglycemia/none-hyperglycemia.	40%	7.12	224.9	
Patients with CVD	14%	3.4	56.3	<0.001
Free CVD	0.35	0.18	0.67	
Obesity/none-obesity.	64.7%	7.4	565.9	
Patients with CVD	24.3%	3.4	171.6	<0.001
Free CVD	0.37	0.21	0.65	
MetS/none -MetS	35%	6.1	199.9	
Patients with CVD	13.7%	3.3	56.1	<0.001
Free CVD	0.39	0.20	0.74	
Smoking/none-smoking.	55%	8.8	343.2	
Patients with CVD	16.4%	4.1	66.3	<0.001
Free CVD	0.29	0.13	0.68	
Hypertension/none-hypertension.	31.7%	6.16	162.6	
Patients with CVD	10.8%	2.7	42.5	<0.001
Free CVD	0.34	0.20	0.59	

## DISCUSSION

This study aimed to assess the metabolic and cardiovascular risk characteristics among pre-D compared to T2DM and control groups and emphasizing key factors that could influence disease advancement and cardiovascular issues, focusing on the evaluation of biochemical, anthropometric and lifestyle factors. The outcomes show significant metabolic alteration and increased risk for CVD in pre-D, suggesting urgent intervention for early screening and treatment.

The distribution of age and gender among participants was similar, proving that demographic features have no impact on metabolic outcomes. However, the significant variation in educational attainment across individuals suggests that a reduced level of education may be associated with a greater risk of metabolic problems, either as a result of lifestyle choices, lack of health literacy, or financial limitations. This finding is consistent with previous research suggesting that reduced education correlates with a higher risk of metabolic syndrome and adverse health outcomes.^8^,^9^

A markedly increase in BMI, FM and F% in pre-D and T2DM groups compared to controls suggests that body fat is an essential factor in metabolic imbalance, this agrees with previous knowing evidence: high body fat, particularly visceral fat, contributes to insulin resistance and overall inflammation.^10^,^11^ The incidence of T2DM was marked by gradual increase in FPG, HbA1c and insulin levels and these markers showed statistical significance (p<0.001). Individuals with prediabetes might exhibit increased insulin levels resulting from compensatory hyperinsulinemia linked to insulin resistance, typically arising before apparent β-cell dysfunction.^12^ Moreover, TG levels were considerably elevated in the T2DM group (p = 0.001), supporting the atherogenic dyslipidemia typically observed in diabetic individuals in previous findings.^13^

In the present study, smoking and hypertension were linked to disturbances in metabolic profiles; patients with pre-D and T2DM who smoked showed increased LDL and TC levels, whereas those with hypertension had notably higher systolic and diastolic blood pressures, reflecting the combined effect of these factors on worsening cardiovascular risk.^14^

Insulin strongly correlated with BMI and FM, strengthening the association between fatness and insulin resistance. Obesity is characterized by elevated fat mass, which leads to growth and multiplication (hyperplasia) of adipocyte cells.^15^ An excess fat intake beyond what the body requires leads to storage as triglycerides, which often accumulate in visceral and subcutaneous turns to be harmful leading to adipose tissue dysfunction and restricted hyperplastic remodeling, finally causing adipocyte hypertrophy and systemic metabolic problems. Unhealthy hypertrophic obesity associated with abdominal fat has a significant bad impact on metabolic health, attracting macrophages and other immune cells thus leading to systemic inflammation. Obesity is well known has highly correlation with insulin resistance.^16^ Moreover, the rise in fatty acids related to obesity can induce insulin resistance via intracellular metabolites that activate of serine/threonine kinases, that obstruct insulin signaling.^17^

Regression analysis determined that BMI, F%, FM, FPG and insulin serve as independent predictors of HbA1c levels in individuals with pre-D. Our results showed that high glycemic levels in prediabetes are closely associated with body composition and insulin behavior rather than traditional cardiovascular factors such as blood pressure or cholesterol. This explains the importance of regular measurement of body fat in addition to BMI and WC, during initial evaluations of metabolic risk.^18^,^19^

The odds ratio of selected factors associated with CVD collectively shows that hyperglycemia, obesity, metabolic syndrome, smoking and hypertension are all significantly and independently linked to a heightened risk of disease among individuals with prediabetes. The notable differences in odds between cardiovascular and non-cardiovascular groups highlight the necessity for tailored prevention approaches that concentrate on these modifiable risk factors in people with prediabetes, previous findings support our research.^1^,^20^

Our findings challenge the commonly held belief that prediabetes is a relatively harmless condition. Instead, prediabetes refers to a period of subtle but significant metabolic deterioration during which cardiovascular problems emerge. This emphasizes the importance of implementing preventive strategies such as lifestyle management, medical treatment and structured early monitoring during progression of the illness.^20^,^21^

### Limitations

Among limitations, cross-sectional design cannot determine causation and low sample size might impact statistical power. Future research needed using longitudinal designs should investigate the time-dependent relationship between these variables.

## CONCLUSION

Pre-D subjects showed serious metabolic problems and cardiovascular risks. Adiposity-related variables, particularly FM and F%, have significant effect on glycemic progression, whereas smoking, hypertension and obesity significantly elevate cardiovascular risk. These results support a change in approach towards earlier, comprehensive intervention in the prediabetic

### Authors’ Contribution:

**AMA:** Statistical analysis, interpretation, writing, final approval and responsible and accountable for the accuracy of the study.

**MAA, AMA, and AAA:** Statistical analysis, Literature review, and drafting.

**FSA and AJA:** Data collection and Critical analysis.

All authors have read and approved the final version of the manuscript.
